# Sympathetic nerve-derived ATP regulates renal medullary vasa recta diameter via pericyte cells: a role for regulating medullary blood flow?

**DOI:** 10.3389/fphys.2013.00307

**Published:** 2013-10-29

**Authors:** C. Crawford, S. S. P. Wildman, M. C. Kelly, T. M. Kennedy-Lydon, C. M. Peppiatt-Wildman

**Affiliations:** Urinary System Physiology Unit, Medway School of Pharmacy, The Universities of Kent and GreenwichChatham Maritime, Kent, UK

**Keywords:** pericytes, ATP release, P2 receptors, vasa recta, vasoconstriction, kidney slice

## Abstract

Pericyte cells are now known to be a novel locus of blood flow control, being able to regulate capillary diameter via their unique morphology and expression of contractile proteins. We have previously shown that exogenous ATP causes constriction of vasa recta via renal pericytes, acting at a variety of membrane bound P2 receptors on descending vasa recta (DVR), and therefore may be able to regulate medullary blood flow (MBF). Regulation of MBF is essential for appropriate urine concentration and providing essential oxygen and nutrients to this region of high, and variable, metabolic demand. Various sources of endogenous ATP have been proposed, including from epithelial, endothelial, and red blood cells in response to stimuli such as mechanical stimulation, local acidosis, hypoxia, and exposure to various hormones. Extensive sympathetic innervation of the nephron has previously been shown, however the innervation reported has focused around the proximal and distal tubules, and ascending loop of Henle. We hypothesize that sympathetic nerves are an additional source of ATP acting at renal pericytes and therefore regulate MBF. Using a rat live kidney slice model in combination with video imaging and confocal microscopy techniques we firstly show sympathetic nerves in close proximity to vasa recta pericytes in both the outer and inner medulla. Secondly, we demonstrate pharmacological stimulation of sympathetic nerves *in situ* (by tyramine) evokes pericyte-mediated vasoconstriction of vasa recta capillaries; inhibited by the application of the P2 receptor antagonist suramin. Lastly, tyramine-evoked vasoconstriction of vasa recta by pericytes is significantly less than ATP-evoked vasoconstriction. Sympathetic innervation may provide an additional level of functional regulation in the renal medulla that is highly localized. It now needs to be determined under which physiological/pathophysiological circumstances that sympathetic innervation of renal pericytes is important.

## Introduction

Dogma suggests that arterioles and precapillary sphincters are the key regulatory mechanisms of blood flow at the tissue level, however it is now becoming widely accepted that an additional locus of local blood flow regulation exists—that being pericyte cells found on the capillaries themselves (Peppiatt-Wildman, 2013). Pericyte cells are smooth muscle-like cells, comprised of a cell body and long processes, which run along and wrap-around the capillary, and possess the contractile proteins α-smooth muscle actin and myosin. Pericytes have the ability to constrict and dilate capillaries, via their processes, following exposure to a number of vasoactive agents including adenosine 5′-triphosphate (ATP) (Peppiatt et al., 2006; Crawford et al., 2012; Peppiatt-Wildman, 2013).

Pericytes have been identified on capillaries in many organs of the body, including the kidney. Within the kidney, pericytes are primarily located on descending vasa recta (DVR) capillaries (Park et al., 1997). DVR extend from the renal cortex toward the outer and then inner medulla, with the highest density of pericytes being observed in the outer medulla (Park et al., 1997; Crawford et al., 2012). It has been suggested that renal pericytes play an integral role in regulating renal medullary blood flow (MBF), which in itself is essential to satisfy the conflicting demands of preserving the cortico-medullary gradients of NaCl and urea, while maintaining adequate oxygen and nutrient delivery, as well as metabolic clearance (Kennedy-Lydon et al., 2013). As such it has been further proposed that dysregulation and/or dysfunction of the pericyte cells themselves may account and/or contribute to a number of renal pathologies (Crawford et al., 2012; Peppiatt-Wildman, 2013).

Using our live tissue slice model (i.e., where vasa recta and pericytes are located *in situ*), we previously demonstrated that exogenous ATP, a well-documented vasoactive transmitter, causes constriction of vasa recta via renal pericytes (Crawford et al., 2011). We further demonstrated that exogenous noradrenaline (NA) causes constriction of DVR via renal pericytes (Crawford et al., 2012), and ATP acts via a variety of pericyte-membrane bound P2 receptors (*nee* purinoceptors), (Crawford et al., 2011). Consequently, this study added to an already substantial wealth of scientific publications that propose key physiological roles for extracellular ATP and P2 receptors (i.e., purinergic signaling) in the kidney; specifically in this case, a key role for purinergic signaling in regulating renal MBF (Crawford et al., 2011).

Given the increasing interest in renal purinergic signaling it is perhaps not surprising that various sources of endogenous extracellular ATP have been proposed, including renal epithelial, endothelial and red blood cells, in response to stimuli such as: mechanical stimulation (e.g., stretch, increased flow rate, and osmotic swelling), local acidosis, hypoxia, exposure to various hormones (e.g., vasopressin and aldosterone), and to a lesser extent from sympathetic nerve varicosities (Bodin et al., 1991; Sprague et al., 1996; Schwiebert, 2001; Jans et al., 2002; Burnstock and Knight, 2004; Praetorius et al., 2005; Wildman et al., 2009).

Extensive sympathetic innervation of the kidney has previously been shown, however the innervation reported has focused around the proximal tubules, distal tubules and ascending loop of Henle (Dibona, 2000). Sympathetic nerves follow the arterial vasculature from the renal artery to the outer medullary portions of the descending (and ascending) vasa recta, but have not been reported in the inner medulla or papilla regions (Barajas et al., 1992). Electron microscopy has demonstrated that sympathetic nerve varicosities come into close contact with effector cell membranes (both tubular and vascular cell) in the renal cortex, where released transmitters presumably act on receptors expressed by the effector cells. It is likely that a balance between sympathetic nerves and intra-renal effector cells is essential for optimal renal function (Dibona, 2005). Both ATP and NA are co-released from sympathetic nerve varicosities (Burnstock, 1990). Sympathetic nerve stimulation has been shown to induce the release of ATP and NA in human cortical kidney slices (Rump et al., 1996). *In vivo* studies in rats and rabbits have shown that cortical blood flow (CBF) and total renal blood flow (RBF) are regulated in part, by renal sympathetic nerves, whereby electrical stimulation causes constriction of arteries and arterioles at higher frequencies (Rudenstam et al., 1995; Leonard et al., 2000). We now know quite a lot about the effect of sympathetic nerve stimulation on MBF (Walkowska et al., 2005; Eppel et al., 2006a,b; Johns et al., 2011) but some important questions remain, for example are vasa recta pericytes innervated, can neurotransmitter release from nerves associated with said pericytes induce vasoconstriction, and does ATP contribute to this vasoconstriction?

Given that renal pericytes are likely key regulators of MBF, and that purinergic (and noradrenergic) signaling potentially plays a role in the regulatory process, we hypothesize that sympathetic nerves may be an additional source of ATP acting at renal pericytes to regulate MBF. In the current study we use our novel kidney tissue slice model in combination with immunohistochemistry to determine the proximity of vasa recta pericytes to sympathetic nerves in the renal medulla. In addition we investigate the effect of pharmacologically stimulating the release of transmitter substances (ATP and NA) from sympathetic nerves on pericyte activity. Identification of a new pathway (i.e., sympathetic innervation) for the regulation of renal pericytes may inform studies seeking to delineate the elusive mechanisms underlying renal pathology.

## Methods

### Tissue slicing

Animal experiments were conducted in accordance with United Kingdom Home Office Scientific Procedures Act (1986). Kidney tissue slices were obtained as previously described (Crawford et al., 2012). Adult male Sprague Dawley rats (~250 g, purchased from Charles River UK Ltd., Kent, UK) were killed by cervical dislocation; their kidneys were immediately removed and placed in ice-cold physiological saline solution (PSS) bubbled with 95% O_2_/5% CO_2_ and prepared for slicing. Prior to slicing, the kidneys were de-capsulated and any renal artery remnants removed. A single kidney was secured on the slicing block of a vibratome tissue slicer (Leica model VT1200S, Leica Microsystems (UK) Ltd, Milton Keynes, UK), and submerged in a bath of ice cold PSS bubbled with 95% O_2_/5% CO_2_. PSS contained (mM) 100 NaCl, 5 KCl, 0.24 NaH_2_PO_4_, 0.96 Na_2_HPO_4_, 10 Na acetate, 1 CaCl_2_, 1.2 MgSO_4_, 5 glucose, 25 NaHCO_3_, 5 pyruvate (Sigma-Aldrich Ltd, Dorset, UK). The pH was adjusted to 7.4 using 10 M NaOH. The outer cortical dome region (~3 mm tissue) of the kidney was removed to expose the top of the renal medulla and serial 200 μm-thick coronal kidney slices (in which there was intact cortex and medulla) were cut. Slices were collected and maintained at room temperature in a holding chamber containing PSS, and bubbled with 95% O_2_/5% CO_2_ to conserve tissue viability. The slices to be used in “live” experiments were maintained for up to 3 h in the holding chamber, or slices were fixed with 4% paraformaldehyde (PFA, Sigma-Aldrich Ltd, Dorset, UK) and used for immunohistochemistry.

### Immunohistochemistry

For experiments in which pericytes and sympathetic nerves were co-labeled, fixed kidney tissue slices were incubated with anti-NG2 (Calbiochem, Merck Millipore, Middlesex, UK) and anti-tyrosine hydroxylase (anti-TH; Vector Laboratories Ltd, Peterborough, UK) primary antibodies, respectively. The anti-NG2-labeled pericytes were probed with a fluorescent Alexa Fluor 555-conjugated secondary antibody (Invitrogen Life Technologies, Paisley, UK), as previously described (Crawford et al., 2012). The tyrosine-hydroxylase signal was amplified with a biotinylated secondary antibody and probed with a FITC-conjugated tertiary antibody (Fluorescent Avidin Kit, Vector Laboratories Ltd, Peterborough, UK). Kidney slices were mounted using Citiflour (Agar Scientific Ltd., Stanstead, UK). The medulla of fixed slices was imaged using a Zeiss LSM 510 laser scanning confocal microscope (Carl Zeiss Ltd., Welwyn Garden City, Hertfordshire, UK). FITC-conjugated secondary antibodies were excited at 488 nm and Alexa Fluor 555-conjugated secondary antibody excited at 543 nm. Emitted light was collected with the following filters: long-pass 560 nm (Alexa Fluor 555) and band-pass 505–550 nm (FITC). Pericytes and sympathetic nerves were imaged in both the inner and outer medulla. Z-stack images (40× magnification) were also obtained in order to measure the distance between nerves and pericytes. At least three z-stacks were obtained in both inner and outer medulla per slice. The distances between sympathetic nerves and the nearest pericyte, in both inner and outer medulla, were measured using Zeiss LSM image browser software (Carl Zeiss Ltd., Welwyn Garden City, Hertfordshire, UK).

### Functional experiments and analysis

Functional DIC imaging experiments in live tissue, investigating *in situ* pericyte-mediated changes in vasa recta diameter, were performed as previously described (Crawford et al., 2011, 2012). Live kidney slices were secured in an open-bath chamber using a purpose-built platinum slice anchor and transferred to the stage of an upright Olympus microscope (model BX51WI, Olympus Microscopy, Southend-on-Sea, UK). Kidney slices were continuously superfused (~2.5 ml/min; 1.25 ml bath volume) with PSS, bubbled with 95% O_2_/5% CO_2_ and maintained at room temperature. Pericytes on the vasa recta capillaries (vasa recta were defined as <10 μm in diameter) were identified by their previously established “bump-on-a-log” morphology (Crawford et al., 2012), and DIC images were captured through an Olympus 60× water immersion objective (0.9 NA). Real-time video images of changes in vasa recta diameter were collected every second by an attached Rolera XR Charge Coupled Devise (CCD) camera and recorded using Image Pro Software (Media Cybernetics Inc., Marlow, UK). Kidney slices were superfused with PSS alone for 70 s to establish a baseline vessel diameter at the pericyte and non-pericyte site. Slices were then exposed to tyramine [1 μ M; for ~150 s, sufficient time to evoke neurotransmitter release (Glover et al., 1983)], ATP (100 μ M), suramin (100 μ M), or combinations of the agents, before being subjected to a PSS wash. Compounds were purchased from Sigma-Aldrich Ltd, Dorset, UK, and working solutions were prepared in oxygenated PSS.

Time-series analysis of live kidney slice experiments was carried out using the public domain software ImageJ (NIH, http://rsb.info.nih.gov.ij), as previously described (Crawford et al., 2012). For each experiment, both a pericyte site and a non-pericyte site were identified on a single vasa recta and the diameter of the vasa recta at both locations was measured every five frames for the duration of the experiment (1 frame = 1 s). Each capillary acted as its own control; an average of the first five measurements was taken to represent the resting baseline diameter value, and expressed as 100%, for both pericyte and non-pericyte sites. All subsequent diameter measurements were calculated and expressed as a percentage of the corresponding baseline value for both pericyte and non-pericyte sites as previously described (Crawford et al., 2012).

For all experiments, statistical significance was calculated using Student's *t*-test; *P* < 0.05 (two tailed) was considered significant. Values are expressed as mean ± SEM; sample size (*n*) represents the number of pericytes (1 pericyte and non-pericyte site per kidney slice). All experiments were performed in at least three different animals. One vasa recta per kidney slice was used to ensure all vessels were “naïve” prior to exposure to any drug.

## Results

### Sympathetic innervation of the renal medulla

**Figure 1:**
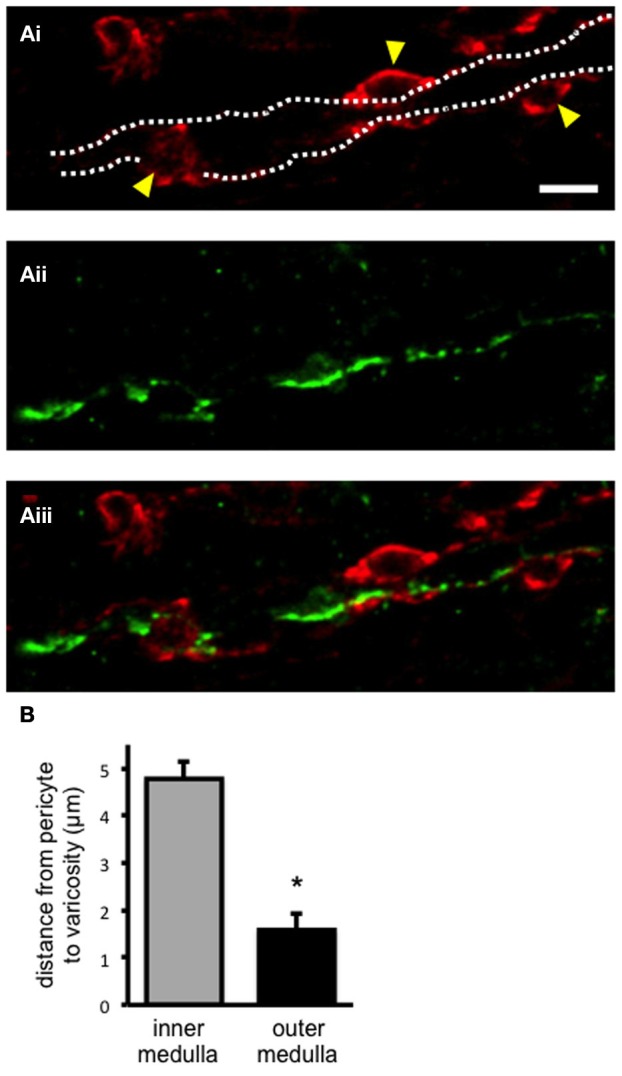
**Sympathetic nerves in close apposition to pericyte cells in the renal medulla**. In **(Ai)**, confocal image of fixed kidney slices (outer medulla region) incubated with anti-NG2, and probed with Alexa 555-conjugated secondary antibody (red) to label vasa recta pericytes and their processes. Pericyte cell bodies are highlighted (arrowheads), and the capillary wall is outlined (dotted lines). In **(Aii)**, from the same image as in **(Ai)**, sympathetic nerves were labeled with anti-tyrosine hydroxylase primary antibody, probed with Alexa 488-conjugated secondary antibody (green). In **(Aiii)**, an overlay of images **(Ai, Aii)** shows sympathetic nerves running along vasa recta capillaries, in close proximity to pericytes. Scale bar = 10 μm. The distance between pericytes and the nearest sympathetic nerve varicosity was measured from confocal images using image browser software. In **(B)**, the distance between pericyte and varicosity was significantly less in the outer medulla region (*n* = 21) when compared to the inner medulla (*n* = 21; ^*^P < 0.05).

Pericytes and sympathetic nerves were labeled in the medulla of fixed kidney tissue slices using anti-NG2 and tyrosine hydroxylase (TH) antibodies, respectively, as described in the methods section (Figures 1Ai, Aii). Pericytes were clearly defined by their bump-on-a-log morphology (Figure 1Ai). Sympathetic nerves were consistently found running through the entire medulla region [i.e., both the outer (*n* = 21) and inner medulla (*n* = 19)]. In all slices analyzed, sympathetic nerves were found, irrespective of localization in the medulla, to be in close proximity to vasa recta perciytes (Figure 1Aiii). Confocal z-stack images were used to follow nerves through the tissue, and the distance from pericyte to the nearest sympathetic nerve varicosity (defined by their nodule-like appearance) was measured using image browser software (see Supplementary Movie 1 for an example z-stack image). The distance from pericyte to the nearest sympathetic nerve varicosity was significantly less in the outer medulla (1.58 ± 0.35 μm, *n* = 21) compared to the inner medulla (4.78 ± 1.08 μm, *n* = 19, ^*^P < 0.05, Figure 1B). Appropriate, parallel control experiments were carried out omitting anti-TH, anti-NG2, and/or biotinylated secondary antibodies, which confirmed an absence of fluorescent labeling.

### Effect of tyrosine-evoked neurotransmitter release on vasa recta diameter

**Figure 2:**
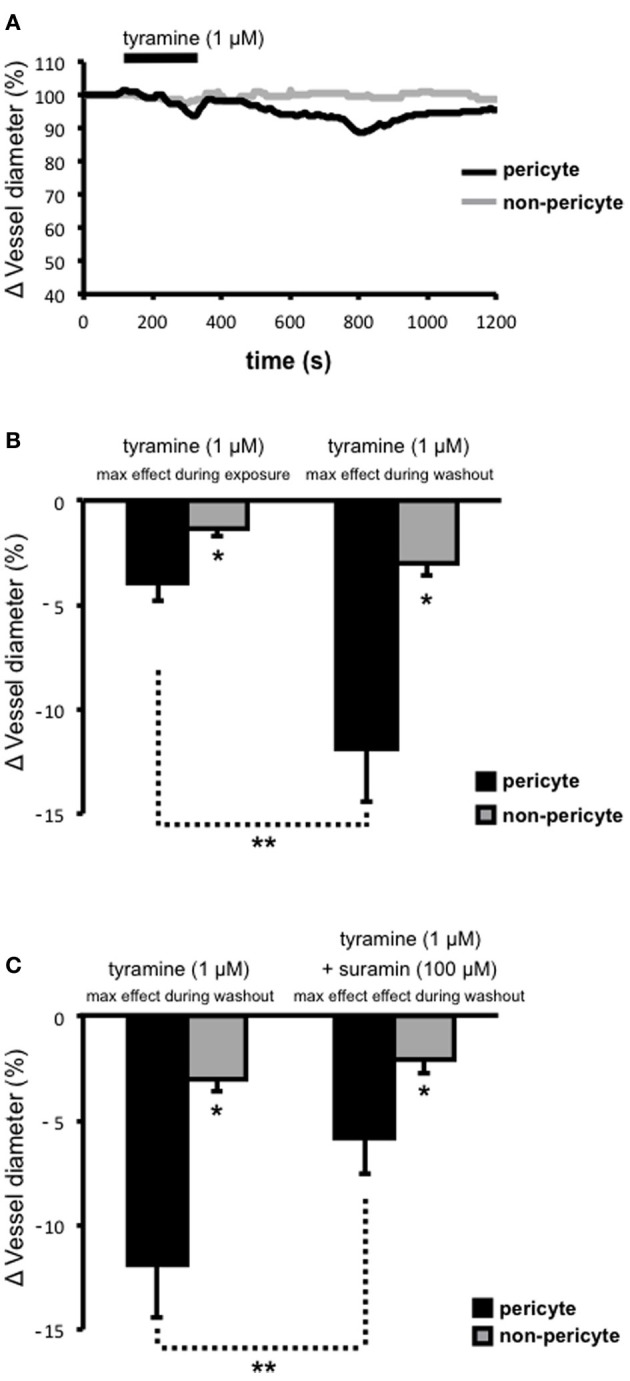
**Tyramine-evoked changes in vasa recta diameter**. Live kidney slices were superfused with tyramine (1 μM). A typical trace from a single experiment shows changes in vessel diameter over time at a pericyte (**A**, black line) and corresponding non-pericyte site (**A**, gray line). Mean data in bar graphs in **(B)**, and **(C)**, show a significantly greater change in vessel diameter at pericyte sites (black bars) compared to non pericyte sites (gray bars), both during tyramine (1 μM) exposure and during the tyramine-washout period (**B**, ^*^P < 0.05, *n* = 7). The constriction measured at pericyte sites was significantly greater during the tyramine washout step than that measured when tyramine was present in the bath (**B**, ^**^P < 0.05, *n* = 7). The maximum tyramine-evoked constriction seen at pericyte sites was significantly inhibited by the presence of suramin (**C**, 100 μM, ^**^P < 0.05, *n* = 6).

Tyramine was superfused onto live kidney tissue slices in order to stimulate the release of sympathetic neurotransmitters (i.e., ATP and NA; via two distinct mechanisms; displacement from the axoplasm and subsequent vesicular release) from varicosities (Trendelenberg et al., 1987; Kirkpatrick and Burnstock, 1994) and the ability of tyramine-evoked neurotransmitter release to alter the diameter of subsurface vasa recta at both pericyte and non-pericyte sites was measured. Superfusion (~150 s) of tyramine (1 μ M) caused a slowly-reversible vasoconstriction of vasa recta at pericyte sites (Figure 2A). During exposure to tyramine, vasoconstriction was significantly greater at pericyte sites (3.9 ± 0.9%, *n* = 7) than at non-pericyte sites (1.4 ± 0.3%, *n* = 7, ^*^P < 0.05, Figure 2B). Maximum tyramine-evoked vasoconstriction occurred ~550 s (534 ± 115 s, *n* = 7) after the cessation of tyramine superfusion (i.e., during tyramine-washout, Figure 2A). Maximum vasoconstriction, i.e., during tyramine-washout, at the pericyte site was 11.9 ± 2.5% as compared to 3.0 ± 0.6% at the non-pericyte site (*n* = 7, ^*^P < 0.05, Figure 2B), and was significantly greater than that measured when tyramine was present in the bath (^**^P < 0.05, *n* = 7, Figure 2B). Complete reversibility (i.e., when vessel diameter returned to baseline) was seen ≥600 s from the start of tyramine-washout (*n* = 7). Using Poiseuille's law, the maximum tyramine-evoked vasoconstriction of 12%, equates to a ~1.7-fold increase in vasa recta resistance and thus a ~40% decrease in blood flow (Peppiatt et al., 2006; Crawford et al., 2012).

Tyramine evokes the co-release of ATP and NA from sympathetic nerve varicosities. To investigate the proportion of ATP-mediated vasoconstriction *vs*. NA-mediated vasoconstriction, suramin was superfused onto the tissue slices, for the entirety of each experiment, in order to antagonize ATP-activated P2 receptors, including those present on renal pericytes. Superfusion (for ~150 s) of tyramine (1 μ M) following suramin pre-treatment, and in the continued presence of suramin (100 μ M), resulted in the maximum tyramine-evoked vasoconstriction (measured during tyramine-washout) being reduced by approximately 50% at pericyte sites (^**^P < 0.05; Figure 2C). The maximum tryamine-evoked vasoconstriction at pericyte sites, in the presence of suramin, was reduced to 5.8 ± 1.7%, and was still significantly greater than the change in vessel diameter recorded at non-pericyte sites (2.1 ± 0.7%, ^*^P < 0.05, *n* = 6; Figure 2C). Incidentally, in 6 out of 10 experiments suramin (100 μ M) alone caused a modest vasoconstriction of vasa recta at pericyte sites (2–5%). Whilst suramin significantly altered the maximum tyramine-evoked constriction of vasa recta by pericytes, the presence of suramin did not alter the onset time, time to maximum effect, nor reversibility, of tyramine-evoked vasoconstriction (data not shown, *n* = 6).

**Figure 3:**
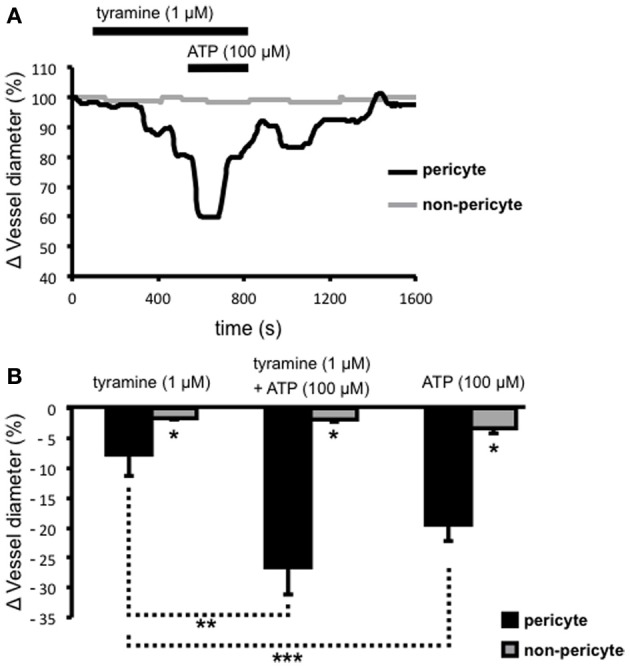
**The additive effects of ATP and tyramine on pericyte-mediated changes in vasa recta diameter**. Live kidney slices were superfused with tyramine (1 μM) and ATP (100 μM); a typical trace from one of these experiments shows changes in vessel diameter over time at a pericyte (**A**, black line) and corresponding non-pericyte site (**A**, gray line). Mean data for these experiments show a significantly greater change in vessel diameter at pericyte sites (black bars) compared with non-pericyte sites (gray bars) under all experimental conditions (^*^P < 0.05, *n* = 5). The combined tyramine (1 μM) and ATP (100 μM)-evoked constriction at pericyte sites was significantly greater than that observed during tyramine (1 μM) incubation alone (**B**, ^**^P < 0.05, *n* = 5), and the pericyte-mediated constriction of vasa recta in the presence of exogenous ATP was significantly greater than that evoked by tyramine alone (^***^P < 0.05, *n* > 5).

Extracellular ATP acts at pericytes to cause vasoconstriction of vasa recta (Crawford et al., 2011) and various sources of endogenous extracellular ATP have been proposed including from renal epithelial cells, endothelial cells, red blood cells, and sympathetic nerve varicosities. To investigate the significance/relative contribution of varicosity-derived ATP to ATP-evoked, pericyte-mediated, vasoconstriction of vasa recta, the additive effects of tyramine and ATP (100 μ M), at a concentration previously shown to evoke the greatest constriction of vasa recta (Crawford et al., 2011), were investigated. Prolonged superfusion (~800 s) of tyramine (1 μ M) caused a constriction of vasa recta at pericyte sites (Figure 3A) culminating in a maximum effect between 400–600 s (*n* = 5). During exposure to tyramine, maximum vasoconstriction was significantly greater at pericyte sites (7.8 ± 3.6%, *n* = 5) than at non-pericyte sites (1.6 ± 0.3%, *n* = 5, ^*^P < 0.05, Figure 3B). The co-application of ATP (100 μ M; for ~200 s) with tyramine (1 μM) during the “maximum tyramine-evoked vasoconstriction period” caused a further increase (^**^P < 0.05, Figure 3B) in the constriction of vasa recta at pericyte sites (26.5 ± 4.6%) that was significantly greater than at non-pericyte sites (1.7 ± 0.5%, *n* = 5, ^*^P < 0.05, Figure 3B). The exogenous ATP-evoked vasoconstriction rapidly desensitized in the presence of exogenous ATP and complete reversibility was seen ≥700 s from the start of tyramine/exogenous ATP-washout (Figure 3A). Superfusion (for ~150 s) of ATP (100 μ M) alone caused a reversible vasoconstriction of vasa recta at pericyte sites; maximum exogenous ATP-evoked vasoconstriction at the pericyte site was 19.4 ± 2.8% as compared to 3.3 ± 2.9% at the non-pericyte site (^*^P < 0.05, *n* = 11, Figure 3B). The exogenous application of ATP (100 μ M) alone caused a significantly greater constriction than that observed with tyramine, at pericyte sites (1 μ M, ^***^P < 0.05, Figure 3B).

## Discussion

**Figure 4:**
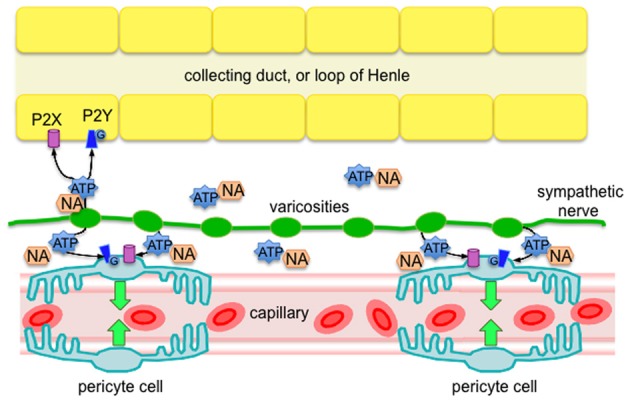
**Diagram of proposed sympathetic innervation of renal pericytes in the outer medulla, whereby ATP is a key neurotransmitter, along with NA, released from sympathetic nerve varicosities**. Released ATP is likely to act via P2 receptors (ionotropic P2X, or metabotropic P2Y) on pericyte cells to evoke localized vasoconstriction of vasa recta capillaries. Based on the findings of others (Loesch et al., 2009), it is likely that epithelial cells of the outer medulla also receive sympathetic innervation that affect an abundance of P2 receptors (Wildman et al., 2008), further supporting the phenomenon of pericyte-mediated tubulovascular crosstalk in the renal medulla (Peppiatt-Wildman, 2013).

The main findings of this investigation, using the live kidney slice model, revealed sympathetic nerves in both the outer and inner medulla regions of the kidney, and that pharmacological stimulation of sympathetic nerves *in situ* (by tyramine) evokes pericyte-mediated vasoconstriction of vasa recta capillaries. More specifically, we present evidence that (i) sympathetic nerve varicosities are in closer apposition to pericytes in the outer medulla, (ii) tyramine-evoked vasoconstriction of vasa recta by pericytes is significantly inhibited by the P2 receptor antagonist suramin, (iii) tyramine-evoked vasoconstriction of vasa recta by pericytes is significantly less than exogenous ATP-evoked vasoconstriction. Importantly, these novel findings reveal innervation of renal pericytes by sympathetic nerves, primarily in the outer medulla. This finding being ratified by previous studies, which describe innervation of medullary tubules (Dibona, 2000; Loesch et al., 2009). Moreover, functionally these findings demonstrate that sympathetic nerve-derived ATP, acting as a cotransmitter, is as effective as, or more effective than NA, in its ability to regulate vasa recta diameter, and thus MBF. As such, sympathetic innervation may provide an additional level of functional regulation in the renal medulla that is highly localized (see Figure 4).

### Sympathetic nerve varicosities in close apposition to pericytes in the outer medulla

The current study identifies, for the first time, TH-labeled, sympathetic varicosities in both the outer and inner medulla regions of fixed rat kidney tissue (Figure 1). Our novel live kidney slice model in combination with confocal z-sectional imaging enabled us to observe the path of sympathetic nerves through the tissue, running in parallel to vasa recta capillaries in the renal medulla (see Supplementary Movie 1). Furthermore, we demonstrate that sympathetic nerve varicosities are in close apposition to pericyte cells on vasa recta (Figure 1). Interestingly, sympathetic nerve varicosities are in closer apposition to pericytes in the outer medulla rather than inner medulla (Figure 1B), perhaps supporting a less important role for sympathetic innervation of vasa recta pericytes in the inner medulla where their density is known to be significantly less (Crawford et al., 2012). Pericyte cells were found to be ~1.5 μm from the nearest varicosity in the outer medulla, as opposed to ~5 μm in the inner medulla. Autonomic nervous system synaptic clefts, which allows delivery of co-transmitters to the site of the effector cells, vary in size with clefts size ranging from 10 nm up to 2 μm (Burnstock, 2008). Varicosities being similar structures, whereby co-transmitters are released “en passage,” likely affect effector cells in a 2 μm proximity. Given the close proximity (~1.5 μm) of sympathetic nerve varicosities to pericytes in the outer medulla, it is therefore plausible that co-transmitters released by apposite sympathetic nerve varicosities could act at contractile vasa recta pericytes to regulate vasa recta capillary diameter and thus MBF, as hypothesized. Similarly, anti-TH antibodies have previously been used to identify sympathetic nerve varicosities in close apposition to proximal tubule and collecting duct epithelial cells of the rat kidney cortex (Loesch et al., 2009). Authors propose that sympathetic neurotransmitters (primarily ATP), released from varicosity vesicles, may regulate tubule function in the renal cortex through the activation of a variety and abundance of ATP-activated P2 receptors; shown to be expressed throughout the nephron by others (Wildman et al., 2009). In accordance with the functional data describing a key role for sympathetic nerve-derived ATP in the regulation of vasa recta diameter via contractile pericytes, we have previously reported that vasa recta and associated pericytes express mRNA for P2X1, 3, and 7 and P2Y4 and 6 (Crawford et al., 2011). Interestingly, *in vivo* studies in rabbits conclude that P2X receptors do not contribute to neurally mediated vasoconstriction (Eppel et al., 2006a,b,c), however this is perhaps not surprising given that we have previously demonstrated much higher levels (40-fold) of P2Y (P2Y_4_ and P2Y_6_) than P2X receptor mRNA in rat isolated vasa recta, with pericytes *in situ* (Crawford et al., 2011); supporting sympathetically-derived ATP acting via P2Y receptors on pericytes to mediate vasoconstriction of vasa recta.

It is perhaps interesting to note that whilst all capillary networks are deemed to have a degree of pericyte coverage, capillaries in skeletal muscle of the rat (Saltzman et al., 1992), and peripheral blood vessels of the mouse (Long and Segal, 2009), are not innervated; suggesting that not all pericytes receive sympathetic innervation.

### ATP is a key neurotransmitter released from sympathetic varicosities

As well as being a substrate for NA synthesis, the ability of tyramine to evoke sympathetic neurotransmitter release is well documented (Kirkpatrick and Burnstock, 1994). Notably in our preparation, the time between application of tyramine and maximum dilation of vasa recta by pericytes (~550 s) is greater than that seen by the superfusion of a vasoactive agent like NA or ATP (~100 s; Crawford et al., 2011, 2012). In our slice preparation it is likely that tyramine is both responsible for release, synthesis and subsequent release of newly synthesized co-transmitters (Figure 2A). Various neurotransmitters, most notably NA and ATP are contained in a single sympathetic nerve varicosity, all of which are released at one time, the proportion of each released in the current study was unknown. Pharmacological intervention, using a specific ATP-activated P2 receptor antagonist, suramin, allowed an estimation of the proportion of the tyramine-evoked response attributable to ATP released from sympathetic nerves. Tyramine-evoked vasoconstriction of vasa recta by pericytes was significantly inhibited (~50%) by the P2 receptor antagonist suramin (Figure 2C), suggesting a key role for sympathetic purinergic signaling in the regulation of MBF by renal pericytes. Interestingly, others have shown that the α-adrenoceptor antagonist prazosin greatly reduces RBF and CBF in response to renal nerve stimulation, yet MBF was both reduced and increased (Chapman et al., 1982; Eppel et al., 2004, 2006a,b,c). This may of course be due to the relative expression of α-adrenoceptors in the vasculature throughout the kidney, which was not reported by authors of these studies.

### Sympathetic innervation provides a fine-tuning mechanism

The current study demonstrates that the vasoconstriction of vasa recta by pericytes evoked by: (i) superfusion of ATP onto live kidney slices, and (ii) tyramine-stimulated release of co-transmitters, are additive. The renal medulla contains numerous potential sources of vasoactive ATP, including from tubular cells, endothelial cells, and red blood cells (Bodin et al., 1991; Sprague et al., 1996; Schwiebert, 2001; Jans et al., 2002; Praetorius et al., 2005; Wildman et al., 2009), in addition to that released by sympathetic nerve varicosities. The additive effects of neuronal co-transmitters and paracrine-released ATP on the renal vasculature, has previously been demonstrated to enhance smooth-muscle cell-mediated vasoconstriction of the glomerular afferent arteriole (Hultstrom et al., 2007). That tyramine-evoked constriction of vasa recta by pericytes is significantly less than exogenous ATP-evoked effects, or indeed maximal exogenous NA-evoked effects (Crawford et al., 2011, 2012), to our minds suggest that sympathetic innervation fine-tuning of MBF can be achieved. However, it is noteworthy that different proportions of co-transmitter are released in different tissues and the contribution of each co-transmitter can depend on a number of parameters of stimulation, e.g., that short bursts of electrical stimulation favor ATP release, and longer bursts favor NA (Burnstock, 2007).

## Conclusions

We hypothesized that sympathetic nerves are an additional source of ATP to act upon renal pericytes to therefore regulate MBF (see Figure 4). Here we present evidence to support our hypothesis; however acknowledge that *in vivo* studies are still required to provide direct information regarding the control of MBF. What remains to be determined is under which physiological and/or pathophysiological circumstances that sympathetic innervation of renal pericytes, and therefore MBF, is important. Like others we assume that coordination exists between sympathetic nerves and intra-renal effectors such as vessels, tubules and juxtaglomerular cells [neatly termed tubulovascular crosstalk (Dickhout et al., 2002; Kennedy-Lydon et al., 2013; Peppiatt-Wildman, 2013), see Figure 4], and that there are functionally specific subgroups of renal nerve fibers mediating specific effects on the renal tubular, vascular or juxtaglomerular granular cells, such as those involved in RBF response and urinary flow rate response (Dibona et al., 1996; Dibona, 2000). A better understanding of these neural sub-populations will determine the significance of renal purinergic innervation and MBF, which is undoubtedly overlooked in light of renal transplantation (denervated kidney) successes, and misconception that kidneys have complete intrinsic ability to regulate blood flow without the need for autonomic input [despite it being well accepted that control of renal hemodynamics involves both intrinsic (myogenic and TGF components of autoregulation) and extrinsic mechanisms]. To contextualize the potential significance of these findings, considering what is now known about the regulation of MBF: despite the hypoxic environment of the renal medulla, regulated MBF serves to provide the vascular and highly metabolic tubular cells with adequate oxygen and nutrients whilst clearing metabolic waste. Imbalances in MBF regulation are detrimental to the health of the kidney as a whole, with localized ischemia leading to papillary necrosis and loss of appropriate sodium and water transport in the loops of Henle. Moreover, it is well established that dysregulation of MBF, acute or chronic, can result in significant pathology. Should sympathetically-derived ATP be involved in said dysregulation, renal pericytes, and indeed purinergic signaling pathways, may represent a novel a therapeutic target in the future.

### Conflict of interest statement

The authors declare that the research was conducted in the absence of any commercial or financial relationships that could be construed as a potential conflict of interest.

## Supplementary material

Supplementary Movie 1 **Z-stack series of images showing co-localization of pericytes and sympathetic nerves in the outer medulla**. Pericytes and sympathetic nerves were labeled with anti-NG2, and tyrosine hydroxylase primary antibodies, respectively, and were subsequently probed with fluorescent Alexa-555 (red) and Alexa-488 (green)-conjugated antibodies, respectively. The z-stack series images 5.6 μm through the tissue slice; sympathetic nerves can be seen to run in close proximity to pericytes on vasa recta capillaries. Scale bar = 10 μm.

## References

[B1] Barajas L. Liu L. Powers K. (1992). Anatomy of the renal innervation: intrarenal aspects and ganglia of origin. Can. J. Physiol. Pharmacol. 70, 735–749 10.1139/y92-098 1423018

[B2] Bodin P. Bailey D. Burnstock G. (1991). Increased flow-induced ATP release from isolated vascular endothelial cells but not smooth muscle cells. Br. J. Pharmacol. 103, 1203–1205 10.1111/j.1476-5381.1991.tb12324.x 1652343 PMC1908098

[B3] Burnstock G. (1990). Noradrenaline and ATP as cotransmitters in sympathetic nerves. Neurochem. Int. 17, 357–368 10.1016/0197-0186(90)90158-P 20504636

[B4] Burnstock G. (2007). Physiology and pathophysiology of purinergic neurotransmission. Physiol. Rev. 87, 659–797 10.1152/physrev.00043.2006 17429044

[B5] Burnstock G. (2008). Non-synaptic transmission at autonomic neuroeffector junctions. Neurochem. Int. 52, 14–25 10.1016/j.neuint.2007.03.007 17493707

[B6] Burnstock G. Knight G. E. (2004). Cellular distribution and functions of P2 receptor subtypes in different systems. Int. Rev. Cytol. 240, 31–304 10.1016/S0074-7696(04)40002-3 15548415

[B7] Crawford C. Kennedy-Lydon T. M. Callaghan H. Sprott C. Simmons R. L. Sawbridge L. (2011). Extracellular nucleotides affect pericyte-mediated regulation of rat *in situ* vasa recta diameter. Acta Physiol. (Oxf.) 202, 241–251 10.1111/j.1748-1716.2011.02310.x 21624094

[B8] Crawford C. Kennedy-Lydon T. M. Sprott C. Desai T. Sawbridge L. Munday J. (2012). An intact kidney slice model to investigate vasa recta properties and function *in situ*. Nephron Physiol. 120, 17–31 10.1159/000339110 22833057 PMC5166522

[B9] Chapman B. J. Horn N. M. Robertson M. J. (1982). Renal blood-flow changes during renal nerve stimulation in rats treated with alpha-adrenergic and dopaminergic blockers. J Physiol. 325, 67–77 6125590 10.1113/jphysiol.1982.sp014136 PMC1251380

[B10] Dibona G. F. (2000). Neural control of the kidney: functionally specific renal sympathetic nerve fibers. Am. J. Physiol. Regul. Integr. Comp. Physiol. 279, R1517–R1524 11049831 10.1152/ajpregu.2000.279.5.R1517

[B11] Dibona G. F. (2005). Physiology in perspective: the wisdom of the body. Neural control of the kidney. Am. J. Physiol. Regul. Integr. Comp. Physiol. 289, R633–R641 10.1152/ajpregu.00258.2005 16105818

[B12] Dibona G. F. Sawin L. L. Jones S. Y. (1996). Differentiated sympathetic neural control of the kidney. Am. J. Physiol. 271, R84–R90 8760207 10.1152/ajpregu.1996.271.1.R84

[B13] Dickhout J. G. Mori T. Cowley A. W. Jr. (2002). Tubulovascular nitric oxide crosstalk: buffering of angiotensin II-induced medullary vasoconstriction. Circ. Res. 91, 487–493 10.1161/01.RES.0000035243.66189.92 12242266

[B14] Eppel G. A. Lee L. L. Evans R. G. (2004). alpha-Adrenoceptor subtypes mediating regional kidney blood flow responses to renal nerve stimulation. Auton. Neurosci. 112, 15–24 10.1016/j.autneu.2004.03.001 15233926

[B15] Eppel G. A. Ventura S. Denton K. M. Evans R. G. (2006a). Lack of contributionof P2X receptors to neurally mediated vasoconstriction of the rabbit kidney *in vivo*. Acta Physiol. 186, 197–207 10.1111/j.1748-1716.2006.01526.x 16497199

[B16] Eppel G. A. Ventura S. Evans R. G. (2006b). Regional vascular responses to ATP and ATP analogues in the rabbit kidney *in vivo*: roles for adenosine receptors and prostanoids. Br. J. Pharmacol. 149, 523–531 10.1038/sj.bjp.0706901 16981003 PMC2014670

[B17] Eppel G. A. Luff S. E. Denton K. M. Evans R. G. (2006c). Type 1 neuropeptide Y receptors and alpha1-adrenoceptors in the neural control of regional renal perfusion. Am. J. Physiol. Regul. Integr. Comp. Physiol. 290, R331–R340 10.1152/ajpregu.00317.2005 16195497

[B18] Glover V. Pycock C. J. Sandler M. (1983). Tyramine-induced noradrenaline release from rat brain slices:prevention by (-)-deprenyl. Br. J. Phramacol. 80, 141–148 10.1111/j.1476-5381.1983.tb11059.x 6418254 PMC2044979

[B19] Hultstrom M. Lai E. Y. Ma Z. Kallskog O. Patzak A. Persson A. E. (2007). Adenosine triphosphate increases the reactivity of the afferent arteriole to low concentrations of norepinephrine. Am. J. Physiol. Regul. Integr. Comp. Physiol. 293, R2225–R2231 10.1152/ajpregu.00287.2007 17928513

[B20] Jans D. Srinivas S. P. Waelkens E. Segal A. Lariviere E. Simaels J. (2002). Hypotonic treatment evokes biphasic ATP release across the basolateral membrane of cultured renal epithelia (A6). J. Physiol. 545, 543–555 10.1113/jphysiol.2002.026641 12456833 PMC2290701

[B21] Johns E. J. Kopp U. C. DiBona G. F. (2011). Neural control of renal function. Compr. Physiol. 1, 731–767 10.1002/cphy.c100043 23737201

[B22] Kennedy-Lydon T. M. Crawford C. Wildman S. S. Peppiatt-Wildman C. M. (2013). Renal pericytes: regulators of medullary blood flow. Acta Physiol. 207, 212–225 10.1111/apha.12026 23126245 PMC3561688

[B23] Kirkpatrick K. A. Burnstock G. (1994). Release of endogenous ATP from the vasa deferentia of the rat and guinea-pig by the indirect sympathomimetic tyramine. J. Auton. Pharmacol. 14, 325–335 10.1111/j.1474-8673.1994.tb00613.x 7829537

[B24] Leonard B. L. Evans R. G. Navakatikyan M. A. Malpas S. C. (2000). Differential neural control of intrarenal blood flow. Am. J. Physiol. Regul. Integr. Comp. Physiol. 279, R907–R916 10956248 10.1152/ajpregu.2000.279.3.R907

[B25] Loesch A. Unwin R. Gandhi V. Burnstock G. (2009). Sympathetic nerve varicosities in close apposition to basolateral membranes of collecting duct epithelial cells of rat kidney. Nephron Physiol. 113, p15–p21 10.1159/000235246 19684415

[B26] Long J. B. Segal S. S. (2009). Quantifying perivascular sympathetic innervation: regional differences in male C57BL/6 mice at 3 and 20 months. J. Neurosci. Methods 184, 124–128 10.1016/j.jneumeth.2009.07.028 19651158 PMC2761756

[B27] Park F. Mattson D. L. Roberts L. A. Cowley A. W. Jr. (1997). Evidence for the presence of smooth muscle alpha-actin within pericytes of the renal medulla. Am. J. Physiol. 273, R1742–R1748 9374818 10.1152/ajpregu.1997.273.5.R1742

[B28] Peppiatt C. M. Howarth C. Mobbs P. Attwell D. (2006). Bidirectional control of CNS capillary diameter by pericytes. Nature 443, 700–704 10.1038/nature05193 17036005 PMC1761848

[B29] Peppiatt-Wildman C. M. (2013). The evolving role of renal pericytes. Curr. Opin. Nephrol. Hypertens. 22, 10–16 10.1097/MNH.0b013e32835b4e6e 23165111

[B30] Praetorius H. A. Frokiaer J. Leipziger J. (2005). Transepithelial pressure pulses induce nucleotide release in polarized MDCK cells. Am. J. Physiol. Renal Physiol. 288, F133–F141 10.1152/ajprenal.00238.2004 15367389

[B31] Rudenstam J. Bergstrom G. Taghipour K. Gothberg G. Karlstrom G. (1995). Efferent renal sympathetic nerve stimulation *in vivo*. Effects on regional renal haemodynamics in the Wistar rat, studied by laser-Doppler technique. Acta Physiol. Scand. 154, 387–394 10.1111/j.1748-1716.1995.tb09922.x 7572236

[B32] Rump L. C. Bohmann C. Schwertfeger E. Krumme B. Von Kugelgen I. Schollmeyer P. (1996). Extracellular ATP in the human kidney: mode of release and vascular effects. J. Auton. Pharmacol. 16, 371–375 10.1111/j.1474-8673.1996.tb00056.x 9131419

[B33] Saltzman D. Delano F. A. Schmid-Schonbein G. W. (1992). The microvasculature in skeletal muscle. VI. Adrenergic innervation of arterioles in normotensive and spontaneously hypertensive rats. Microvasc. Res. 44, 263–273 10.1016/0026-2862(92)90086-5 1479927

[B34] Schwiebert E. M. (2001). ATP release mechanisms, ATP receptors and purinergic signalling along the nephron. Clin. Exp. Pharmacol. Physiol. 28, 340–350 10.1046/j.1440-1681.2001.03451.x 11339211

[B35] Sprague R. S. Ellsworth M. L. Stephenson A. H. Lonigro A. J. (1996). ATP: the red blood cell link to NO and local control of the pulmonary circulation. Am. J. Physiol. 271, H2717–H2722 8997335 10.1152/ajpheart.1996.271.6.H2717

[B36] Trendelenberg U. Langeloh A. Bonisch H. (1987). Mechanism of action of indirectly acting sympathomimetic amines. Blood Vessels 24, 261–270 10.1159/000158702 3620709

[B37] Walkowska A. Badzyñska B. Kompanowska-Jezierska E. Johns E. J. Sadowski J. (2005). Effects of renal nerve stimulation on intrarenal blood flow in rats with intact or inactivated NO synthases. Acta Physiol. Scand. 183, 99–105 10.1111/j.1365-201X.2004.01376.x 15654923

[B38] Wildman S. S. Kang E. S. King B. F. (2009). ENaC, renal sodium excretion and extracellular ATP. Purinergic Signall. 5, 481–489 10.1007/s11302-009-9150-6 19306075 PMC2776138

[B39] Wildman S. S. Marks J. Turner C. M. Yew-Booth L. Peppiatt-Wildman C. M. King B. F. (2008). Sodium-dependent regulation of renal amiloride-sensitive currents by apical P2 receptors. J. Am. Soc. Nephrol. 19, 731–742 10.1681/ASN.2007040443 18235098 PMC2390963

